# Multiple insecticide resistance mechanisms involving metabolic changes and insensitive target sites selected in anopheline vectors of malaria in Sri Lanka

**DOI:** 10.1186/1475-2875-7-168

**Published:** 2008-08-28

**Authors:** M Devika B Perera, Janet Hemingway, SHP Parakrama Karunaratne

**Affiliations:** 1 Regional Office, Anti Malaria Campaign, Kurunegala, Sri Lanka; 2 Vector Research Group, Liverpool School of Tropical Medicine, Pembroke Place, Liverpool, L3 5QA, UK; 3 Department of Zoology, Faculty of Science, University of Peradeniya, Peradeniya, Sri Lanka

## Abstract

**Background:**

The current status of insecticide resistance and the underlying resistance mechanisms were studied in the major vector of malaria, *Anopheles culicifacies*, and the secondary vector, *Anopheles subpictus *in five districts (Anuradhapura, Kurunegala, Moneragala, Puttalam and Trincomalee) of Sri Lanka. Eight other anophelines, *Anopheles annularis, Anopheles barbirostris, Anopheles jamesii, Anopheles nigerrimus, Anopheles peditaeniatus, Anopheles tessellatus, Anopheles vagus *and *Anopheles varuna *from Anuradhapura district were also tested.

**Methods:**

Adult females were exposed to the WHO discriminating dosages of DDT, malathion, fenitrothion, propoxur, λ-cyhalothrin, cyfluthrin, cypermethrin, deltamethrin, permethrin and etofenprox. The presence of metabolic resistance by esterase, glutathione S-transferase (GST) and monooxygenase-based mechanisms, and the sensitivity of the acetylcholinesterase target site were assessed using synergists, and biochemical, and metabolic techniques.

**Results:**

All the anopheline species had high DDT resistance. All *An. culicifacies *and *An. subpictus *populations were resistant to malathion, except *An. culicifacies *from Kurunegala, where there was no malathion carboxylesterase activity. Kurunegala and Puttalam populations of *An. culicifacies *were susceptible to fenitrothion. All the *An. culicifacies *populations were susceptible to carbamates. Both species were susceptible to the discriminating dosages of cypermethrin and cyfluthrin, but had different levels of resistance to other pyrethroids. Of the 8 other anophelines, only *An. nigerrimus *and *An. peditaeniatus *were resistant to all the insecticides tested, probably due to their high exposure to the insecticides used in agriculture. *An. vagus *showed some resistance to permethrin.

Esterases, GSTs and monooxygenases were elevated in both *An. culicifacies *and *An. subpictus*. AChE was most sensitive to insecticides in Kurunegala and Trincomalee *An. culicifacies *populations and highly insensitive in the Trincomalee *An. subpictus *population.

**Conclusion:**

The complexity of the resistance segregating in these field populations underlines the need for new molecular tools to identify the genomic diversity, differential upregulation and different binding specificities of resistance conferring genes, and the presence of different subspecies with different vectorial capacities.

## Background

Malaria has been a major public health problem in Sri Lanka since ancient times. Several major malaria epidemics have occurred on the island. The most devastating of these was the epidemic in 1934/35 with more than five million cases and 80,000 deaths. Incidence of malaria declined to an extremely low level during 1958–1963 as a result of the introduction of DDT. However, there was a resurgence of the disease in 1965 leading to a major epidemic in 1968 [1,2]. More recently, several outbreaks of malaria have occurred with varying degree of intensity. *Anopheles culicifacies *is the major vector of malaria, while *Anopheles subpictus*, under favourable ecological conditions, acts as a secondary vector [3,4]. Several other anophelines including *Anopheles annularis, Anopheles barbirostris, Anopheles jamesii, Anopheles nigerrimus, Anopheles peditaeniatus, Anopheles tessellatus *and *Anopheles varuna *occur on the island and several of these are potential vectors [5].

Control of malaria in Sri Lanka is primarily through the use of insecticides. Prior to 1975/77 malaria vector control programmes were based on indoor residual house spraying (IRS) of DDT. Due to development of resistance to DDT in vector mosquito populations and environmental concerns, DDT was replaced with malathion in 1975 [2]. Development of vector resistance to malathion, and increased transmission of malaria, lead to the introduction of pyrethroids for vector control programmes in Sri Lanka in 1994. At present, fenitrothion (an organophosphate) and pyrethroids such as λ-cyhalothrin, cyfluthrin, and deltamethrin or the pseudo-pyrethroid etofenprox are the major insecticides used for IRS. Rotation of chemically unrelated compounds every 3–5 years is the strategy adopted by the Sri Lankan malaria vector control programmes to delay the development of resistance. Permethrin (a pyrethroid) is the only insecticide used for impregnation of mosquito nets. Recently, long-lasting insecticide-treated mosquito nets have been distributed in some provinces [6].

Insecticide resistance is increasingly becoming a problem for malaria vector control programmes. Widespread use of the same insecticides in the agricultural sector has made the situation worse. Resistance may develop due to changes in the mosquitoes enzyme systems, resulting in more rapid detoxification or sequestration of the insecticide, or due to mutations in the target site preventing the insecticide-target site interaction [7]. Insecticides that can be used in malaria control are increasingly becoming limited. Introduction of inappropriate insecticides without a proper understanding of the prevailing resistance mechanisms may lead to enhanced vector resistance and disease control failure. Early detection and knowledge on the resistance status and the underlying mechanisms in vector mosquitoes are essential for effective long-term control of the vector. The status of insecticide resistance and prevalence of different types of resistance mechanisms in *An. culicifacies *and *An. subpictus *populations from five administrative districts of Sri Lanka is reported in this paper. Resistance in other anophelines is also discussed.

## Methods

### Collection of *An. culicifacies *and *An. subpictus *mosquitoes

**Figure 1:**
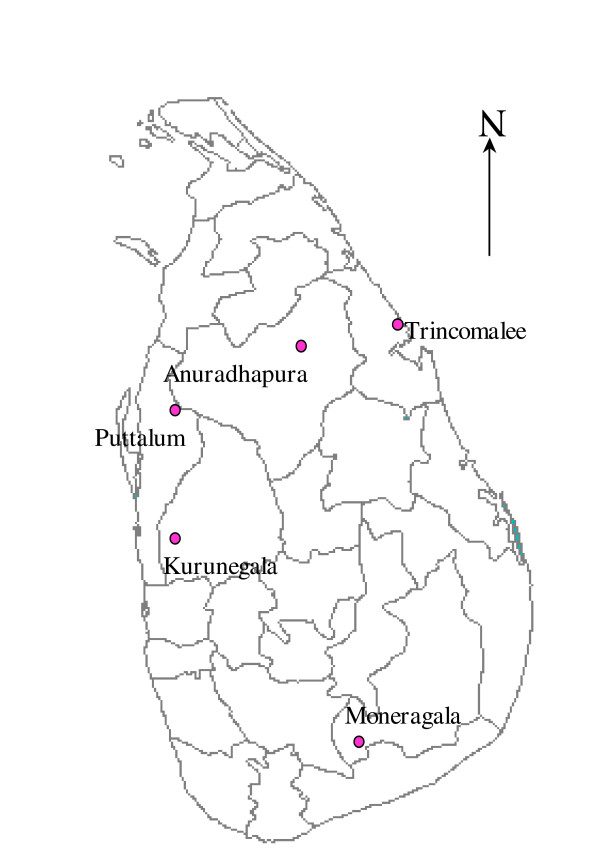
Map of Sri Lanka showing the study sites.

Adult blood-fed female *An. culicifacies *and *An. subpictus *were collected between January 2001–December 2004 using cattle baited hut traps, from selected localities in five administrative districts of Sri Lanka; namely Paderellewa in Anuradhapura district (8°.40 N, 80.74 E°), Gadolwaka in Kurunegala district (7°.67 N, 79.94 E°), Pelwattha in Moneragala district (6°.74 N, 81.19E°), Neelabemma in Puttalam district (8°.19 N, 80.09 E°) and Puliyankulam in Trincomalee district (8°.60 N, 81.20 E°) (Figure 1). F1 adults were obtained from field collected adults. Alternatively, larvae were collected from their natural habitats such as stream bed pools, stream margins, paddy fields and quarry pits and reared through to adults. This procedure allowed standardizing the age and testing conditions. Two-three day old female mosquitoes were then used for all experiments.

### Collection of other anopheline species

*Anopheles annularis, An. barbirostris, An. jamesii, An. nigerrimus, An. peditaeniatus, An. tessellatus, An. vagus *and *An. varuna *were collected from Paderellewa, Pennuma, Konwewa and Perimiyankulama in Anuradhapura district in 2004. Two-three day old female F1 mosquitoes were reared from collected material and used for subsequent experiments.

### Insecticides, chemicals and equipments

Chemicals were purchased from Sigma chemicals U.K. unless otherwise stated. Insecticides (97–99% pure) were from ChemService, UK. Malathion, fenitrothion, propoxur, cyfluthrin, cypermethrin, deltamethrin, etofenprox, λ-cyhalothrin and permethrin were used to prepare insecticide-impregnated papers for bioassay experiments. UV_max _kinetic plate reader and KC_3 _software were from Molecular Devices, Bio-Tek, U.S.A. Gel electrophoresis apparatus and protein assay kit were from BIO-RAD, U.K.

### Preparation of insecticide-impregnated papers and insecticide bioassays

Insecticide-impregnated papers were prepared by the standard World Health Organization method [8]. Insecticide solutions of WHO recommended discriminating dosages were prepared by mixing the technical grade insecticide with a spreading agent. DDT (4%), malathion (5%), fenitrothion (1%) and propoxur (0.1%) solutions were prepared in olive oil. Cyfluthrin (0.15%), cypermethrin (0.1%), deltamethrin (0.025%), etofenprox (0.1%), λ-cyhalothrin (0.1%), and permethrin (0.25%) solutions were made in Dow-Corning 556 silicone fluid. For permethrin, deltamethrin and λ-cyhalothrin former WHO discriminating dosages were used [9], as there is evidence to show that these are sufficient to kill 100% of all insecticide susceptible Sri Lankan vectors [10]. Rectangles of Whatman-No. 1 filter papers (12 cm × 15 cm) were used for insecticide impregnation. Insecticide/oil solution (0.7 ml) was mixed with an equal volume of acetone (0.7 ml) and the mixture was spread uniformly on the filter paper.

Insecticide bioassays were conducted by means of tarsal contact exposure to insecticide-impregnated papers using WHO standard bioassay test kits [8]. Batches of 10–20 female mosquitoes (depending on the availability) were exposed to insecticide impregnated papers for one hour except for fenitrothion papers, for which the exposure time was two hours. Dead mosquitoes were counted after a recovery period of 24 hours. At least five replicates for each insecticide were carried out with each population. Papers impregnated with the carrier alone were used as controls. Results were used only if the mortality in the controls was < 20% and the mortalities were adjusted for using Abbott's formula [11]. WHO classification was used to interpret the results [12].

#### Synergist studies

Involvement of mosquito carboxylesterases and monooxygenases in insecticide resistance was further supported by carrying out synergist studies with 2–3 day old adult females from Moneragala district. Triphenyl phosphate (TPP) (10%) was used as a carboxylesterase inhibitor and piperonyl butoxide (PB) (4%) as a monooxygenase inhibitor. Mosquitoes were first exposed to the synergist impregnated paper for one hour and then exposed to the insecticide (malathion, fenitrothion or permethrin) impregnated paper using WHO bioassay test kits.

#### Biochemical experiments

Biochemical experiments were carried out by using the procedures outlined by WHO [13]. Individual mosquitoes were subjected to protein, esterase, glutathione S-transferase (GST), monooxygenase and acetylcholinesterase (AChE) assays. Adult mosquitoes (n = 200 from each population) were individually homogenized in 150 μl of ice cold distilled water. 50 μl of crude mosquito homogenate was taken for the AChE assay and the remaining homogenate was centrifuged at 13,000 g for two minutes. To obtain specific activities of enzymes, protein concentrations of the individual homogenates were determined by Bio-Rad protein determination. In a microtitre plate well, 10 μl of each homogenate was mixed with 300 μl of BIO-RAD working solution (prepared according manufacturers instructions) and absorbance was read at 630 nm after a five minute incubation at room temperature.

#### Esterase assay

Ten (10) μl of each homogenate was mixed with 200 μl of 1 mM *p*-nitrophenyl acetate (pNPA) working solution (100 mM pNPA in acetonitrile: 50 mM sodium phosphate buffer pH 7.4, 1:99) in a microtitre plate well. The reaction was read immediately at 405 nm for two minutes as a kinetic assay at 21°C. An extinction co-efficient of 6.53 mM^-1 ^(corrected for a path length of 0.6 cm) was used to convert the absorbance values to moles of product. Esterase specific activity per individual was reported as μmol product min^-1 ^mg^-1 ^protein.

#### Glutathione S-transferase (GST) assay

Ten (10) μl of each homogenate was mixed with 200 μl of reduced glutathione (GSH)/1-chloro-2,4 dinitrobenzene (CDNB) working solution [95 parts of 10 mM reduced glutathione (GSH) in 100 mM phosphate buffer pH 6.5 + 5 parts of 63 mM CDNB diluted in methanol] in a microtitre plate well. The reaction was read at 340 nm immediately as a kinetic assay for 5 minutes. An extinction co-efficient of 5.76 mM^-1 ^(corrected for a path length of 0.6 cm) was used to convert absorbance values to moles of product. GST specific activity was reported as CDNB conjugated μmol product min^-1 ^mg^-1 ^protein.

#### Monooxygenase estimation

Twenty (20) μl of homogenate was mixed with 80 μl of potassium phosphate buffer pH 7.2 + 200 μl of 6.3 mM tetramethyl benzidine (TMBZ) working solution [(0.01 g TMBZ dissolved in 5 ml methanol and then in 15 ml of sodium acetate buffer pH 5.0) + 25 μl of 3% (v/v) H_2_O_2 _solution] in a microtitre plate well. After a two hour incubation at room temperature, the plate was read at 630 nm as an end-point assay. This assay does not measure the monooxygenase activity, but titrates the amount of bound haem in the mosquito homogenate. Since haem is present in the active site of monooxygenases, major changes in the amount of monooxygenases produces a measureable increase in haem. By using a standard curve of cytocrome C (which also contains bound haem) a crude estimate of the amount of the monooxygenases present was obtained and expressed as equivalent units of cytocrome P450.

#### Malathion metabolism

Batches (25) of adult mosquitoes were homogenized in 0.5 ml of 25 mM Tris-HCl buffer (pH 7.5) and centrifuged at 13,000 g for 5 minutes. Supernatant was incubated with 300 μM malathion for 2 hrs at room temperature. The samples were then extracted with two volumes of 0.5 ml acidified chloroform and dried under an air current. The extract was re-suspended in 30 μl acidified chloroform and loaded onto a silica gel thin layer chromatography (TLC) plate. The plate was eluted with a mobile phase consisting of *n*-hexane: diethyl ether (1:3). After the run the plate was sprayed with a 0.5% (w/v) 2,6-dibromoquinone 4-chloromide in cyclohexane and left at 100°C for 2 hrs to visualize spots of malathion and its metabolic products. Buffer (0.5 ml), incubated with 300 μM malathion and 300 μM sodium hydroxide (NaOH) was run as a positive control. Buffer (0.5 ml), incubated with the same concentration of malathion, was run as a negative control.

#### Acetylcholinesterase (AChE) assay

Two, 20 μl replicates from each mosquito homogenate were placed in adjacent wells of a microtitre plate. The membrane bound AChE in the mosquito homogenate was solubilized by adding 145 μl of Triton phosphate buffer [1% (v/v) Triton X-100 in 0.1 M phosphate buffer pH 7.8] to each. To one set of homogenates, 25 μl of 0.01 M acetylthiocholine iodide (ASChI) and 10 μl of 0.1 M propoxur solution (2.5 ml 0.1 M ASChI + 10 μl of 0.1 M propoxur in acetone) were added. To the other replicate, 25 μl of ASChI alone was added. The kinetics of the enzyme reaction was continuously monitored at 405 nm for five minutes. Results were expressed as a percentage remaining activity in the inhibited fraction compared to the control (uninhibited) activity.

#### Polyacrylamide gel electrophoresis (PAGE)

Native PAGE was used to visualize the pattern of elevated esterase isozymes present in different populations. A mass homogenate of 25 mosquitoes from each population was made in 250 μl of 50 mM sodium phosphate buffer (pH 7.4). Electrophoresis of 10,000 g supernatants from crude homogenates was performed in 7.5% acrylamide gels in tris/borate buffer pH 8.0 containing 0.2 mM EDTA. Gels were stained for esterase activity with 0.04% (w/v) α- and β-naphthyl acetate and 0.1% (w/v) Fast Blue B dye in 100 mM phosphate buffer (pH 7.4). Purple (α-naphthyl acetate preferred) and/or pink (β-naphthyl acetate preferred) esterase bands were identified and the rate of flow (R_f_) was calculated for each band.

## Results

The results of adult bioassays, conducted with discriminative dosages of selected insecticides for *An. culicifacies *and *An. subpictus *populations from different districts are presented in Table 1.

**Table 1:** Percentage mortalities of *Anopheles culicifacies *and *Anopheles subpictus *populations to different insecticides (n > 100 for each value)

	Anuradhapura		Kurunegala		Moneragala		Puttalam		Trincomalee	
	
	Ac	As	Ac	As	Ac	As	Ac	As	Ac	As
4% DDT	34	20	4	14	62	30	24	47	22	19
5% Malathion	39	39	100	49	47	27	30	23	30	30
1% Fenitrothion	88	60	100	62	60	40	100	48	78	40
0.1% Propoxur	100	92	100	84	100	93	100	85	100	86
0.1% λ-cyhalothrin	100	65	100	58	100	100	100	85	100	100
0.25% Permethrin	50	30	85	65	65	67	87	67	23	25
0.025% Deltamethrin	100	88	100	86	7	84	100	92	97	88
0.1% Cypermethrin	100	100	100	100	100	100	100	100	100	100
0.15% Cyfluthrin	100	100	100	100	100	100	100	100	100	100
0.1% Etofenprox	95	27	89	67	85	62	85	74	90	100

Ac = *An. culicifacies*

As = *An. subpictus*.

Mortality 98%–100% indicates susceptibility, 97%–80% suggests possibility of resistance that needs verification, < 80% indicate resistant [11].

All *An. culicifacies *and *An. subpictus *populations had high levels of resistance to DDT. All *An. culicifacies *populations were highly susceptible to the carbamate propoxur. Mortality percentages observed in all *An. subpictus *populations suggested that the possibility of resistance against carbamates. All populations showed resistance to malathion except for *An. culicifacies *from Kurunegala which was fully susceptible. Possible resistance to fenitrothion was shown by the *An. culicifacies *from Anuradhapura (88% mortality) and all other populations were resistant to fenitrothion (< 80% mortality). Kurunegala and Puttalam *An. culicifacies *populations were fully susceptible to fenitrothion. In *An. subpictus*, fenitrothion resistance ranged from 22%–60% (Table 1).

All the populations were susceptible to the WHO discriminating dosages of cypermethrin and cyfluthrin. Moneragala and Trincomalee both *An. culicifacies *and *An. subpictus *populations were susceptible to λ-cyhalothrin. All *An. subpictus *populations showed possible resistance to deltamethrin (84%–92% mortality). Resistance to permethrin occurred in all populations of both species. *An. subpictus *population from Anuradhapura and both *An. culicifacies *and *An. subpictus *populations from Trincomalee had very high levels of resistance to permethrin. Possible resistance to etofenprox was shown by all *An. culicifacies *populations (mortality percentages varied from 81%–90%) but, all *An. subpictus *populations were resistant to etofenprox (27%–74% mortality) with the exception of the Trincomalee population, which was fully susceptible.

Results of synergist studies are given in Table 2. Pre-exposure to the esterase inhibitor TPP reduced the levels of organophosphate and pyrethroid resistance in both species. The monooxygenase inhibitor PB reduced the level of pyrethroid resistance. In contrast, inhibition of monooxygenases by PB prevented the thionate- to oxon – analogue conversion of organophosphates within the insect increasing the organophosphate resistance. The synergist data shows the importance of esterases and monooxygenases in organophosphate and pyrethroid resistance.

**Table 2:** Percentage mortalities *of Anopheles culicifacies *and *Anopheles subpictus *populations from Moneragala district to malathion, fenitrothion and permethrin after exposure to synergists.

Insecticide/ Synergist	*An. culicifacies * % mortality	*An. subpictus * % mortality
5% Malathion	47	27
10% TPP + 5% Malathion	70.9	96
4% PB + 5% Malathion	8.5	10

1% Fenitrothion	60	40
10% TPP + 1% Fenitrothion	80	68
4% PB + 1% Fenitrothion	40	20

0.25% Permethrin	65	67
10% TPP + 0.25% Permethrin	72	50
4% PB 0.25% + Permethrin	92	96

To obtain esterase, glutathione S-transferase and monooxygenase activity profiles, bar charts were constructed to obtain the percentage of the population (Y-axis) which show different activity levels (X-axis).

Activity profiles (specific activity *vs *percentage population) were obtained for esterases and GSTs for each population of each species (20 activity profiles) and quantity profiles for monooxygenases (equivalent units of cytocrome P450 *vs *percentage population) were also plotted for all the populations (10 quantity profiles). After a through analysis of all the profiles (figures not shown), activity peaks corresponding to homozygous susceptible, heterozygous and homozygous resistance were identified. Discriminating values were then established *i.e. *0.25 μmol/mg/min esterase activity, 0.40 μmol/mg/min GST activity and 0.35 equivalent units of monooxygenase amounts, and the percentages of mosquito populations which show more than these values are presented in Table 3.

**Table 3:** Activity of esterases, glutathione S-transferases and monooxygenases in *Anopheles culicifacies *and *Anopheles subpictus *populations (n > 200 for each value)

	Anuradhapura		Kurunegala		Moneragala		Puttalam		Trincomalee	
	
	Ac	As	Ac	As	Ac	As	Ac	As	Ac	As
Est^1^	52	10	51	18	44	21	30	17	15	06
GST^2^	28	19	13	26	10	43	36	45	60	09
MO^3^	24	38	38	28	56	27	27	20	72	86
MCE^4^	+	+	-	++	+++	+++	+	+	+++	+++

Ac = *An. culicifacies*

As = *An. subpictus*

1. % population having esterase specific activity above 0.25 μmol/mg/min

2. % population having glutathione S-transferase specific activity above 0.40 μmol/mg/min

3. % population having monooxygenase levels above 0.35 equivalent units of cytochrome P_450_

4. malathion carboxylesterase activity as measured by malathion metabolism studies (see text for details)

- no activity ++ – moderate +++ – high

Esterase activities of *An. culicifacies *populations were marginally higher than *An. subpictus *populations. The highest esterase activities were found in Anuradhapura and Kurunegala *An. culicifacies *populations, while the lowest was in *An. subpictus *from Trincomalee. Elevation of esterases was evident from gel electrophoresis studies as well. Native PAGE resolved one elevated esterase band from *An. culicifacies *(R_f _= 0.66) and a different elevated esterase from *An. subpictus *(R_f _= 1.12) and the intensity of the bands were higher in the populations which had higher levels of esterase activities. *Anopheles culicifacies *from Trincomalee had the highest GST activity whereas *An. subpictus *from the same area had the lowest GST activity. Estimates of monooxygenase levels were very high for both *An. culicifacies *and *An. subpictus *from Trincomalee (Table 3).

Malathion metabolism studies were undertaken on samples of both species from all five districts to determine whether a malathion-carboxylesterase resistance mechanism was present in these populations. Malathion was metabolized into mono- and di-acid products by mosquito homogenates prepared from *An. culicifacies *and *An. subpictus *from all the districts, with the exception of *An. culicifacies *from Kurunegala. Level of malathion carboxylesterase activity are given for each population in Table 3.

Biochemical assays were undertaken to determine whether there was any evidence for involvement of an altered AChE enzyme mechanism in the organophosphate and/or carbamate resistance observed in these populations. Standard concentrations of propoxur did not inhibit the AChE activity of most of the individuals in both species. The AChE target site of *An. culicifacies *from Kurunegala had a greater sensitivity than that of other *An. culicifacies *populations (Table 4). Highest levels of insensitivity were shown by *An. subpictus *from Trincomalee with a 70% of homozygous insensitive population according to WHO classification [13]. In general, a higher frequency of heterozygous and homozygous insensitive individuals was found in all the *An. subpictus *populations.

**Table 4:** Presence of insensitive target site acetylcholinesterase (AChE) in *Anopheles culicifacies *and *Anopheles subpictus *populations [homozygous sensitive (SS), heterozygous (RS) and homozygous insensitive (RR) are given according to percentage remaining activity of AChE after insecticide inhibition (WHO, 1998)]

	*An. culicifacies * % population			*An. subpictus * % population		
	SS (< 30%)	RS (30–70%)	RR (> 70%)	SS (< 30%)	RS (30–70%)	RR (> 70%)

Anuradhapura	31	42	27	31	53	16
Kurunegala	70	25	5	22	52	26
Moneragala	27	49	25	22	52	26
Puttalam	54	17	29	40	32	28
Trincomalee	60	13	27	17	13	70

Results of the insecticide bioassays carried out with other anopheline mosquitoes are presented in Table 5. All the species showed more than 40% resistance to DDT. Only *An. nigerrimus *and *An. peditaeniatus *were resistant to most of the insecticides tested. Thirty five percent of the *An. vagus *tested was resistant to permethrin. Biochemical assays revealed that monooxygenases are elevated in *An. nigerrimus, An. peditaeniatus *and *An. vagus *populations (Table 6) (large standard deviations indicate high heterogeneity of populations).

**Table 5:** Percentage mortalities of different anopheline species from Anuradhapura to different insecticides (n > 100 for each value)

	*Anopheles annularis*	*Anopheles barbirostris*	*Anopheles jamesii*	*Anopheles nigerrimus*	*Anopheles peditaeniatus*	*Anopheles tessellatus*	*Anopheles vagus*	*Anopheles varuna*
4% DDT	39	07	29	22	57	52	49	39
5% Malathion	100	100	100	87	88	100	100	100
1% Fenitrothion	100	100	100	60	57	100	100	100
0.1% Propoxur	100	100	100	42	64	100	100	100
0.1% λ-cyhalothrin	100	100	100	57	73	100	100	100
0.25% Permethrin	100	100	100	29	47	100	65	100
0.025% Deltamethrin	100	100	100	58	70	100	100	100
0.1% Cypermethrin	100	100	100	88	100	100	100	100
0.15% Cyfluthrin	100	100	100	80	100	100	100	100
0.1% Etofenprox	100	100	100	70	62	100	100	100

**Table 6:** Mean activity levels of insecticide detoxifying enzymes in *Anopheles nigerrimus, Anopheles peditaeniatus and Anopheles vagus *(n = 200 for each value).

Anopheline species	esterase activity μmol min^-1 ^mg^-1 ^ (± SD)	glutathione S-transferase activity μmol min^-1 ^mg^-1 ^ (± SD)	monooxygenase amount (equivalent units of cytochrome P450) (± SD)
*An. nigerrimus*	0.06 (± 0.07)	0.13 (± 0.10)	0.63 (± 0.41)
*An. peditaeniatus*	0.09 (± 0.08)	0.15 (± 0.16)	0.58 (± 0.52)
*An. vagus*	0.16 (± 0.13)	0.24 (± 0.20)	0.89 (± 0.87)

## Discussion

Prior to 1977, DDT was the insecticide used for malaria vector control programmes in Sri Lanka. This was gradually replaced by malathion, due to island-wide development of vector resistance to DDT and concerns for the environment. DDT resistance in *An. culicifacies *and *An. subpictus *was first detected in 1969 in Sri Lanka [2]. Vector resistance to DDT declined slowly after cessation of its usage, but increased again after 1983 due to a GST-based resistance mechanism, which was first selected by exposure to DDT and subsequently selected by exposure to organophosphates [14,15]. Use of malathion, for nearly three decades, has selected high levels of malathion resistance among mosquito vector populations. When malathion was first introduced in Sri Lanka, a 20 minute exposure to 5% malathion gave 100% mortality in *An. culicifacies *[16]. First survivors for this dosage were detected after two years of malathion spraying in 1979. Resistance to the standard WHO discriminating dosage (5% for one hour) was first observed in 1982. Increased malathion carboxylesterase activity was the major underlying mechanism for malathion resistance in *An. culicifacies*, while oxidases played the major role in *An. subpictus *[16]. However, continuous use of malathion later selected malathion carboxylesterases in *An. subpictus *as well [17]. A study carried out in late 1990s in a rural area of Matale district in Sri Lanka showed that both *An. culicifacies *and *An. subpictus *have developed high levels of resistance to organochlorines and organophosphates with multiple resistance mechanisms [10].

Present study shows that resistance to DDT is common in all anopheline species in Sri Lanka. Malathion resistance is present in all the *An. culicifacies *and *An. subpictus *populations tested, except in *An. culicifacies *from Kurunegala, where no malathion carboxylesterase activity was detected. Malathion carboxylesterases are mutated esterases which have high hydrolase activity against malathion, (which has bulky acid groups), but low hydrolase activity against other organophosphates [18,19]. In Sri Lanka, carbamates are used only in the agricultural sector. Carbamate resistance was detected only among the paddy field breeders *i.e. An. subpictus, An. nigerrimus *and *An. peditaeniatus*, probably as a result of their high exposure to agricultural pesticides [20,21].

High levels of insensitive acetylcholinesterase (RS and RR) was found among the *An. culicifacies *and *An. subpictus *populations indicating a high prevalence of insensitive AChE mechanism in these populations. However, the absence of resistance to carbamate propoxur may suggest that the standard dosage of propoxur, which was established using other anopheline species, is not strong enough to inhibit unaltered AChEs of Sri Lankan *An. culicifacies *and *An. subpictus *populations.

With the exception of *An. nigerrimus*, all anopheline populations were completely susceptible to the WHO discriminating dosages of cypermethrin and cyfluthrin. Selection of a monooxygenases-based resistance mechanism, probably due to heavy exposure to a variety of insecticides used in agriculture, has contributed to insecticide resistance in *An. nigerrimus *and *An. peditaeniatus*. High level of susceptibility to tested organophosphates, carbamates and pyrethroids were observed in *An. annularis, An. barbirostris, An. jamesii, An. tessellatus *and *An. varuna*. Mosquito species other than *An. culicifacies*, *An. subpictus, An. nigerrimus, An. peditaeniatus *and *An. vagus *from Anuradhapura gave 100% mortality to 0.25% permethrin. High susceptibility to the discriminating dosages of other pyrethroids may account for the lack of resistance to some pyrethroids observed in all vector populations despite the presence of at least one broad spectrum pyrethroid resistance mechanism.

Insecticide resistance can be due to selection of changes in insect enzyme systems, leading to rapid detoxification or sequestration of insecticide or due to alterations of the insecticide target site preventing the insecticide-target site interaction. Increased metabolic capacity is usually achieved by increased activity of monooxygenases, GSTs or esterases. Metabolic enzyme genes usually have greater plasticity than insecticide target site genes. Increased enzyme activity can be brought about by gene amplification, upregulation, coding sequence mutations or by a combination of these mechanisms. P450s can mediate resistance to all classes of insecticides. GSTs can mediate resistance to organophosphates, organochlorines and pyrethroids. Esterases can provide resistance to organophosphates, carbamates and pyrethroids which are rich with ester-bonds [22,23]. High genetic diversity has caused broad substrate specificity in insect metabolic enzymes. Isolation and characterization of candidate genes/gene families which are over-expressed in these vector populations will aid future vector control programmes.

Insects acquire target site insensitivity through point mutations. However, only a limited number of changes can decrease insecticide sensitivity without disrupting the normal physiological functions of the target site [7]. The classic leucine to phenylalanine mutation of voltage-gated sodium channel proteins, the target site of DDT and pyrethroids, was found in *An. subpictus *from Anuradhapura [24]. This *kdr *type mutation was similar to that of African *An. gambiae s.s. *[25] indicating an independent origin of the same mutation in two different species which are geographically isolated. This shows the constraints of evolving different mutations without interrupting the physiological role of sodium channel. Insensitivity of the carbamate and organophosphate target site AChE, was detected in almost all the populations of *An. culicifacies *and *An. subpictus*. Mutations in target sites alter its binding affinities to different insecticides depending on the molecular structure of the insecticide [26]. Therefore, altered target sites do not mediate the same level of resistance to all the insecticides belong to a particular group.

Heterogeneity of vector resistance to insecticides may also be due to the presence of sibling species with different insecticide cross-resistance spectra. *An. subpictus *species 'A' is abundant predominantly in inland regions and moderately resistant to organophosphates whereas species 'B' is confined to the coast and resistant to permethrin in Sri Lanka [27,28]. The presence of two *An. culicifacies *sub-species 'B' and 'E' has been reported for Sri Lanka. Identification of cross resistance spectra in different subspecies has become an important issue, as only sub-species 'E' transmits disease [29].

Table 7 and Table 8 summarize the levels of insecticide resistance and the detected underlying mechanisms in *An. culicifacies *and *An. subpictus *populations. Resistance spectra can be correlated with the prevalence of different resistance mechanisms in all the populations. High resistance levels of DDT in all populations probably are due to increased levels of GST enzymes. Resistance to malathion in Anuradhapura, Moneragala, Puttalam and Trincomalee populations is probably due to qualitative and quantitative changes of carboxylesterases. Insensitive target-site AChE has also contributed to organophosphate resistance in these populations. Absence of both qualitatively changed carboxylesterase enzymes and altered AChE mechanism must have resulted high susceptibility in Kurunegala *An. culicifacies *population to malathion and fenitrothion. Levels of resistance to pyrethroids shown by the populations are correlated with their high monooxygenase activity levels [30].

**Table 7:** Insecticide cross-resistance spectra and prevalence of resistance mechanisms in different *An. culicifacies *populations.

	DDT	Pro	Mal	Fen	λ-cy	Per	Del	Cyp	Cyf	Eto	CE	GST	Mono	aAChE	MCE
Anuradhapura	RR	SS	RR	RS	SS	RS	SS	SS	SS	RS	+	++	+	++	++
Kurunegala	RR	SS	SS	SS	SS	RS	SS	SS	SS	RS	+	++	+	+	-
Moneragala	RR	SS	RR	RS	SS	RS	RS	SS	SS	RS	+	+	+	+	++
Puttalam	RR	SS	RR	SS	SS	RS	SS	SS	SS	RS	++	++	+	++	++
Trincomalee	RR	SS	RR	RS	SS	RR	RS	SS	SS	RS	+	+++	++	++	+++

SS = susceptible (< 10 R)

RS = intermediate (10–50% R)

RR = resistant (> .50% R) (According to Herath, 1997)

Pro = propoxur

Mal = malathion

Fen = fenitrothion

λ-cy = λ-cyhalothrin

Del = deltamethrin

Per = permethrin

Cyp = cypermethrin

Cyf = cyfluthrin

Eto = etofenprox

CE = carboxylesterase

GST = glutathione S-transferase

Mono = monooxygenase

MCE = malathion carboxylesterase

aAChE = altered acetylcholinesterase

+ = mechanism present at low level, ++ = mechanism present at moderate level, +++ = mechanism present at high level - = absence of mechanism

**Table 8:** Insecticide cross resistance spectra and prevalence of resistance mechanisms in different *An. subpictus *populations.

	DDT	Pro	Mal	Fen	λ-cy	Per	Del	Cyp	Cyf	Eto	CE	GST	Mono	aAChE	MCE
Anuradhapura	RR	SS	RR	RS	RS	RR	RS	SS	SS	RR	+	+	+	+	++
Kurunegala	RR	RS	RR	RS	RS	RS	RS	SS	SS	RS	+	+	+	++	+++
Moneragala	RR	SS	RR	RR	SS	RS	RS	SS	SS	RS	+	++	+	++	+++
Puttalam	RR	RS	RR	RR	RS	RS	SS	SS	SS	RS	+	++	+	++	+++
Trincomalee	RR	RS	RR	RR	SS	RR	RS	SS	SS	SS	+	++	++	+++	+++

SS = susceptible (< 10 R)

RS = intermediate (10–50% R)

RR = resistant (> .50% R) (According to Herath, 1997)

Pro = propoxur

Mal = malathion

Fen = fenitrothion

λ-cy = λ-cyhalothrin

Del = deltamethrin

Per = permethrin

Cyp = cypermethrin

Cyf = cyfluthrin

Eto = etofenprox

CE = carboxylesterase

GST = glutathione S-transferase

Mono = monooxygenase

MCE = malathion carboxylesterase

aAChE = altered acetylcholinesterase

+ = mechanism present at low level, ++ = mechanism present at moderate level, +++ = mechanism present at high level - = absence of mechanism

Genomic diversity, differential upregulation and different binding specificities of resistance conforming genes and the presence of different subspecies with varying vectorial capacities have imposed severe threats to malaria vector control programmes today. The current situation unquestionably demands rapid development of simple molecular tools which are capable of resolving, in the field, the complexity which occurs at taxonomic and phenotypic levels.

## Conclusion

Genomic diversity, differential upregulation and different binding specificities of resistance conforming genes and the presence of different subspecies with varying vectorial capacities have imposed severe threats to malaria vector control programmes today. The current situation unquestionably demands rapid development of simple molecular tools which are capable of resolving, in the field, the complexity which occurs at taxonomic and phenotypic levels.

## Competing interests

The authors declare that they have no competing interests.

## Authors' contributions

SHPPK and JH conceived and designed the study. MDBP conducted all the experiments. All the authors analysed the data and drafted the manuscript. All authors read and approved the final manuscript.
